# Facile Synthesis of Surface-Modified Hollow-Silica (SiO_2_) Aerogel Particles via Oil–Water–Oil Double Emulsion Method

**DOI:** 10.3390/gels10060380

**Published:** 2024-06-02

**Authors:** Pratik S. Kapadnis, Ki-Sun Nam, Hyun-Young Kim, Hyung-Ho Park, Haejin Hwang

**Affiliations:** 1 Department of Materials Science and Engineering, Inha University, Incheon 22212, Republic of Korea; pratikkapadnis@inha.edu (P.S.K.); skarltjs2@naver.com (K.-S.N.); hllykim9@gmail.com (H.-Y.K.); 2 Department of Materials Science and Engineering, Yonsei University, Seoul 03722, Republic of Korea; hhpark@yonsei.ac.kr

**Keywords:** hollow silica (SiO_2_) aerogel, microdroplets, surfactant, oil–water–oil double emulsion

## Abstract

Due to their high surface area and low weight, silica aerogels are ideally suited for several uses, including drug delivery, catalysis, and insulation. Oil–water–oil (*OWO*) double emulsion is a simple and regulated technique for encasing a volatile oil phase in a silica shell to produce hollow silica (SiO_2_) aerogel particles by using hydrophilic and hydrophobic emulsifiers. In this study, the oil–water–oil (*OWO*) double emulsion method was implemented to synthesize surface-modified hollow silica (SiO_2_) aerogel particles in a facile and effective way. This investigation mainly focused on the influence of the N-hexane-to-water glass (*OW*) ratio (*r*) in the first emulsion, silica (water glass) content concentration (*x*), and surfactant concentration (*s*) variations. Furthermore, surface modification techniques were utilized to customize the aerogel’s characteristics. The X-ray diffraction (XRD) patterns showed no imprints of impurities except SiO_2_. Scanning electron microscopy (SEM) and transmission electron microscopy (TEM) images highlight the hollow microstructure of silica particles. Zeta potential was used to determine particle size analysis of hollow silica aerogel particles. The oil–water–oil (*OWO*) double emulsion approach was successfully employed to synthesize surface-modified hollow silica (SiO_2_) aerogel particles, providing precise control over the particle characteristics. By the influence of the optimization condition, this approach improves the aerogel’s potential applications in drug delivery, catalysis, and insulation by enabling surface modifications.

## 1. Introduction

Aerogels are known as highly porous materials (up to ~90 porosity). Aerogels can serve as thermal super-insulators for their low density (80–200 kg/m^3^), which minimizes heat conduction by having low thermal conductivity [1,2]. Because of this excellence, aerogels are lightweight materials with the most excellent insulation properties. The term “aerogel” arises from the observation that they are produced by exchanging the liquid component of a gel with a gas while maintaining the network structure. Silica aerogels are mainly used in various applications like thermal insulation catalysis, drug delivery, oil spill cleanup, sound insulation, and optical applications [3,4].

Hollow silica (SiO_2_) particles are a type of structure with a unique morphology characterized by a hollow interior surrounded by a solid silica shell, which gives them exceptional properties such as low density, high surface area, and unique thermal, electrical, and optical properties [5]. They have gained significant attention due to their diverse applications in fields such as drug delivery, catalysis, sensing, thermal insulation, and environmental remediation [6].

Hollow silica particles can be loaded with drugs or therapeutic agents and used as carriers for targeted drug delivery. The hollow interior provides space for drug loading, while the shell can protect the drug from premature release and degradation [7]. Furthermore, the controlled pore size of the silica shell also can provide a desirable release rate [8]. The high surface area and porosity of hollow silica particles make them excellent candidates for catalytic applications. They can serve as catalysis supports or catalysts in various chemical reactions [9,10]. 

Hollow silica particles can be functionalized with specific molecules or nanoparticles to create sensors for detecting gases, ions, or biomolecules [11]. The large surface area allows for efficient binding of target analytes, leading to sensitive detection. The low thermal conductivity of silica and the hollow interior structure make hollow silica particles useful as insulating materials in applications where thermal insulation is required [12]. Depending on their size and shape, hollow silica particles can exhibit interesting optical properties, such as photonic effects or light scattering [13]. They can be incorporated into coatings, films, or composites for various optical applications [14].

Several methods have been proposed for synthesizing hollow silica particles, including template-assisted synthesis [15], sol–gel processes [16], and emulsion [17] techniques. The template-assisted approach is the most used fabrication technique. A suitable organic [18,19] or inorganic [20] template that will specify the shape and size of the hollow particles is chosen, and the template is coated with silica precursor materials. After silica shell formation, the template is removed, leaving behind the hollow structure. However, the combustion of organic templates such as polystyrene (PS) and latex produces toxic and greenhouse gases such as carbon monoxide (CO) and carbon dioxide (CO_2_) during the template removal process. Inorganic templates such as silica and calcium carbonate and toxic acids such as hydrochloric or hydrofluoric acid need to dissolve selectively in the case of the inorganic templates. In addition, the template-assisted method requires a long processing time for precursor preparation, template coating, shell formation, and template removal.

The sol–gel process is also known as a well-established technique. This technique offers precise control over the size and morphology of the silica particles [21,22]. In this technique, silica precursors, generally tetraethyl orthosilicate (TEOS), are hydrolyzed and then condensed in the presence of a template, which includes an oil droplet or a polymer sphere [23]. Following the removal of the template, a hollow silica structure with a large surface area and low density making it suitable for drug delivery, catalysis, and thermal insulation is obtained [23,24]. Moreover, the procedure can be time-consuming and might involve the use of toxic chemicals.

An emulsion-based synthesis technique is a simple and generally secure approach to producing a wide range of hollow particles with precisely controlled particle size, shape, composition, and spatial arrangement [25]. Emulsion is a two-phase system that is composed of oil droplets dispersed in an aqueous phase or water droplets distributed in an oil phase [26]. Emulsion droplets have proven to be extremely effective templates for the size and structuring of hollow mesoporous silica particles. Liu et al. applied a water-in-oil emulsion technique combined with ambient pressure drying (APD) to produce titania–silica microspheres [27]. Emulsion droplet templates include water-in-oil, oil-in-water, supercritical CO_2_-in-water [28], water–oil–water, and oil–water–oil emulsion systems [29]. Water droplets stabilized by surfactants in oil solution act as templates for the hollow silica particles. A silica shell was produced by hydrolysis and polymerization between a silica precursor such as tetraethyl orthosilicate (TEOS) dissolved in the oil phase with water droplets on a W/O interface [30].

Our earlier work effectively produced spherical surface-modified silica aerogel particles from water glass using an emulsion polymerization technique [31]. This study demonstrates the facile route for synthesizing hollow silica aerogel particles using the *OWO* double emulsion strategy and solvent exchange processes for surface modification. The influence of different optimizing conditions employed in the synthesis of hollow silica aerogel particles was observed. Specifically, the effect of water-to-oil ratio (*r*), concentration of water glass solution (*x*), and surfactant concentration (*s*) in the solution on the formation of a hollow structure of silica particles was studied. In addition to advancing our understanding of the *OWO* double emulsion technique, this work provides a flexible and efficient method for producing surface-modified hollow silica aerogel particles with specific properties for a variety of applications.

## 2. Results and Discussion

In this study, we aimed to optimize critical parameters, specifically the n-hexane to water glass ratio in the primary O/W emulsion (*r*), the concentration of water glass solution (*x*), and surfactant concentration (*s*), to synthesize hollow silica aerogel particles from an O/W/O double emulsion. Changing the above three parameters will affect the morphology and size of oil droplets and the shell thickness of core (n-hexane)–shell (water glass) droplets. Table 1 in the method & materials section provides an overview of the optimization conditions that result in different particle morphologies and summarizes the findings of this systematic exploration.

### 2.1. Effect of n-Hexane to Water Glass Ratio (r) in the Primary O/W Emulsion

The crucial component of the facile synthesis of hollow silica aerogel particles by the double emulsion method is optimizing the hexane-to-water glass ratio in the primary O/W emulsion. Oil phase and water phase concentrations are quite complicated and heavily influenced by the ratio variations [32]. First, our experimental finding was focused on optimizing the hexane-to-water glass ratio in the primary emulsion to understand its impact on the resulting droplet morphology. As shown in Table 1 above, the ratio variations *r_1_* = 20:60 (mL), *r_2_* = 30:50 (mL), and *r_3_* = 40:40 (mL) was followed by increasing the hexane volume and decreasing the water glass volume.

#### 2.1.1. Optical Microscope Analysis

**Figure 1:**
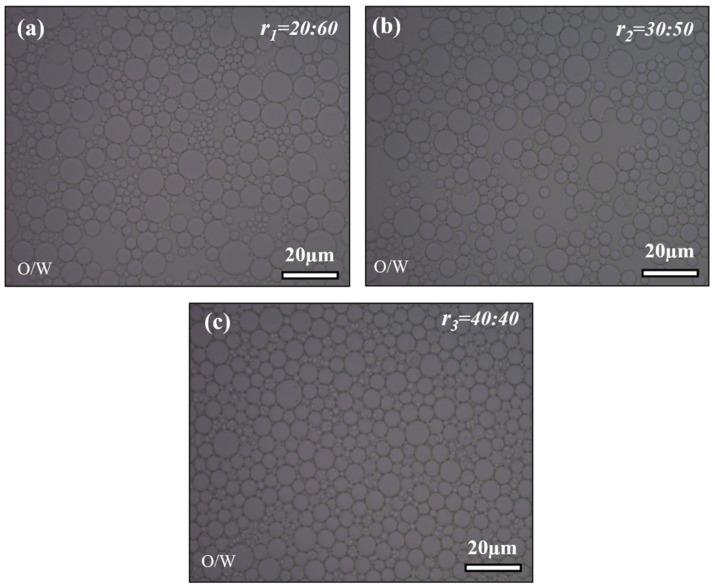
Optical microscopy images: (**a**) *r_1_* = 20:60 (mL), (**b**) *r_2_* = 30:50 (mL), (**c**) *r_3_* = 40:40 (mL) volume ratio for the primary (*OW*) emulsion.

We observed optical microscope images of the primary O/W emulsion to understand the visual representation of the volume ratio effect. As shown in Figure 1 the primary emulsion’s optical microscopy pictures at different volume ratios *r_1_* = 20:60 (mL), *r_2_* = 30:50 (mL), and *r_3_* = 40:40 (mL) provides significant data about the homogeneity of the emulsion, a crucial factor in the O/W/O double emulsion method’s production of hollow-silica aerogel particles. As shown in Figure 1a, the optical microscopy images reveal a lack of homogeneity in oil droplets at the volume ratio of *r_1_* = 20:60 (mL). The irregular and non-uniform distribution of oil droplets indicates difficulties in achieving consistent emulsion, which could be caused by an imbalance between the water and oil phases, resulting in variations in droplet size and distribution.

Moving to the volume ratio *r_2_* = 30:50 (mL) from Figure 1b, the droplet homogeneity is a little improved, although not totally uniform, and some portion of the droplets distributed uniformly, indicating more refined droplets as compared to the *r_1_* = 20:60 (mL). Figure 1c demonstrates that the volume ratio *r_3_* = 40:40 (mL) shows droplets’ uniformity and homogeneous distribution. This ideal emulsion state suggests that an equal distribution of water and oil phases improves the reproducibility of droplet formation. The ratio variations highlight how sensitive the emulsion process is to the balance between the water and oil phases in achieving repeatable and consistent droplet formation.

#### 2.1.2. SEM/TEM Analysis

**Figure 2:**
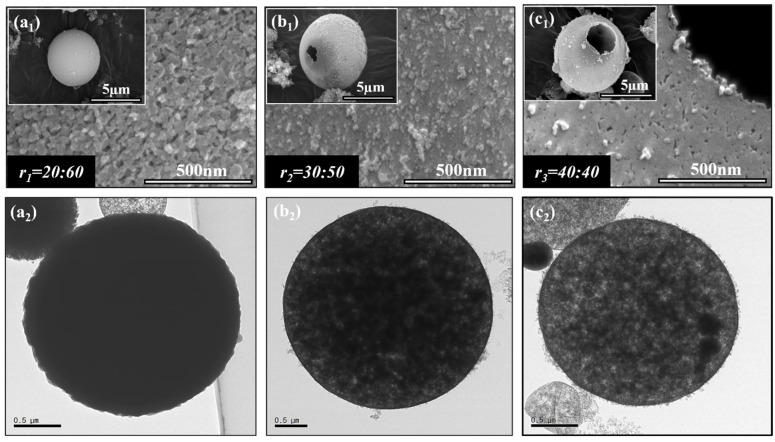
SEM and TEM analysis of silica aerogel particles prepared from (**a_1_**,**a_2_**) *r_1_* = 20:60 (mL), (**b_1_**,**b_2_**) *r_2_* = 30:50 (mL), or (**c_1_**,**c_2_**) *r_3_* = 40:40 (mL) volume ratio (*r*) for the primary (*OW*) emulsion.

Structural and morphological studies of different volume ratio variations *r_1_* = 20:60 (mL), *r_2_* = 30:50 (mL), and *r_3_* = 40:40 (mL) of the primary emulsion were conducted using SEM and TEM observation. A crucial insight into the morphology of the hollow silica aerogel particles synthesized by the *OWO* double emulsion method was demonstrated. Figure 2(a1) indicates that because there was significantly less hexane and a large amount of water glass present in *r_1_*, 20:60 (mL), the particles failed to achieve the hollow morphology, alternatively revealing a porous surface due to the thickness of the silica aerogel core. Furthermore, TEM analysis in Figure 2(a_2_) shows that these microspheres were solid spheres instead of hollow spheres. In contrast, in the SEM image presented in Figure 2(b_1_), slight shifts in particle morphology are observed for the volume ratio *r_2_* = 30:50 (mL). While the hollow structure was formed, the surface characteristics and thickness of the silica shell were noticeable, which confirmed the TEM analysis in Figure 2(b_2_). Figure 2c indicates that the optimized condition *r_3_* = 40:40 (mL) which was observed in the optical microscope image showed the successful synthesis of well-defined spherical hollow silica aerogel particles having a thin, smooth surface. These TEM observations confirm the formation of a hollow core–silica shell structure. Also, they suggest an effective encapsulation of the oil phase within the silica shell. In addition, reducing the hexane impairs the formation of the hollow structure, emphasizing the importance of the *OW* ratio in determining particle morphology.

**Figure 3:**
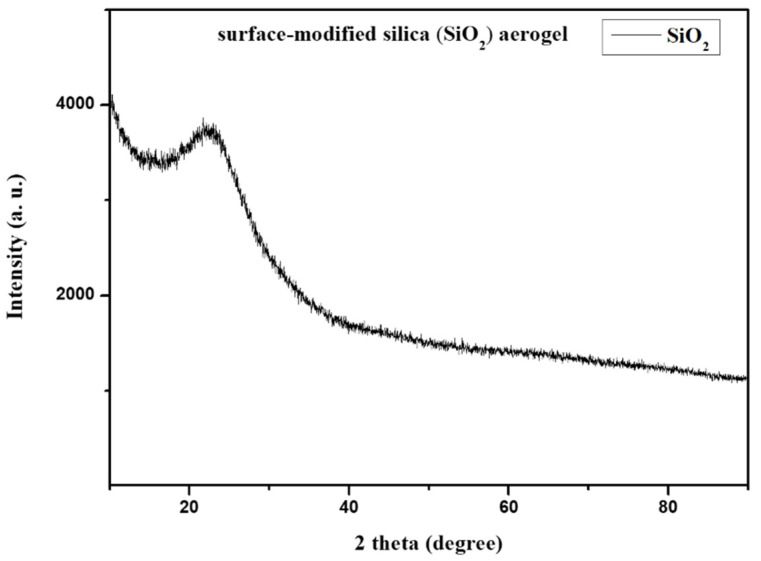
X-ray diffraction (XRD) analysis of surface-modified hollow silica powder.

XRD analysis was conducted for the identification of nature and purity, short-range order information, and structural study. The XRD pattern of hollow silica aerogel powder in Figure 3 exhibits a broad, amorphous peak centered around 2θ = 22°, indicating that silica is non-crystalline and without any impurities [33]. The absence of sharp peaks in the XRD pattern confirms the hollow silica aerogel’s predominantly amorphous structure, which is consistent with the naturally disordered arrangement of atoms within the aerogel network.

### 2.2. Effect of Surfactant [Tween20 and Span80] Concentration [s]

Since the surfactants (tween 20 and span 80) served as both the template for generating mesopores and the interfacial stabilizer for stabilizing the emulsion droplets, the surfactants decreased the interfacial tension between the oil and water phases, stabilizing the droplets and leading to uniformity and smaller droplet size. This stabilization is essential for controlling the shape and size of the silica particles synthesized during the sol–gel process. Higher surfactant concentrations produce smaller and spherical silica particles due to improved stability and prevention of droplet coalescence [34].

#### 2.2.1. Optical Microscope Analysis

To examine the effect of surfactant concentration on droplet morphology, systematic variations were made with the initial emulsifier concentration set at *S_2_* = 1 g for both surfactants (Tween 20 and Span 80). The correlation between the droplet size and surfactant concentration was revealed by optical microscopy analysis. 

**Figure 4:**
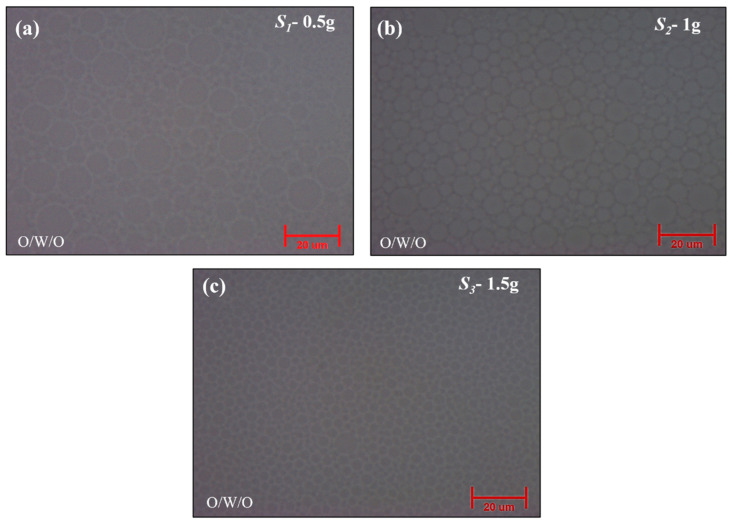
Optical micrographs of different surfactant variations: (**a**) *S_1_* = 0.5 g, (**b**) *S_2_* = 1 g, (**c**) *S_3_* = 1.5 g.

As shown in Figure 4a, larger droplets were seen at lower concentrations of *S_1_* = 0.5 g for Tween 20 and Span 80, which resulted in irregular particle morphologies. Conversely, as shown in Figure 4b, an optimal condition was achieved at the reference concentration of *S_2_* = 1 g for both surfactants, resulting in well-defined and balanced droplets indicative of efficient stabilization during emulsification. Also, as shown in Figure 4c, increasing the surfactant concentration to *S_3_* = 1.5 g resulted in smaller droplets, indicating smaller size droplets but an irregular core formation. The emulsion droplet formation by surfactant concentration variations was used for the synthesis of silica aerogel particles. For the confirmation of the particle size of obtained silica aerogel particles, zeta potential analysis was used, as demonstrated below.

#### 2.2.2. Zeta Potential 

As discussed above, the zeta potential measurements revealed important information about the surface charge of the particles, indicating variations in emulsion stability. Concurrently, particle size analysis confirmed the particle size trends, with larger particles resulting from lower emulsifier concentrations and smaller droplets resulting from higher concentrations.

**Figure 5:**
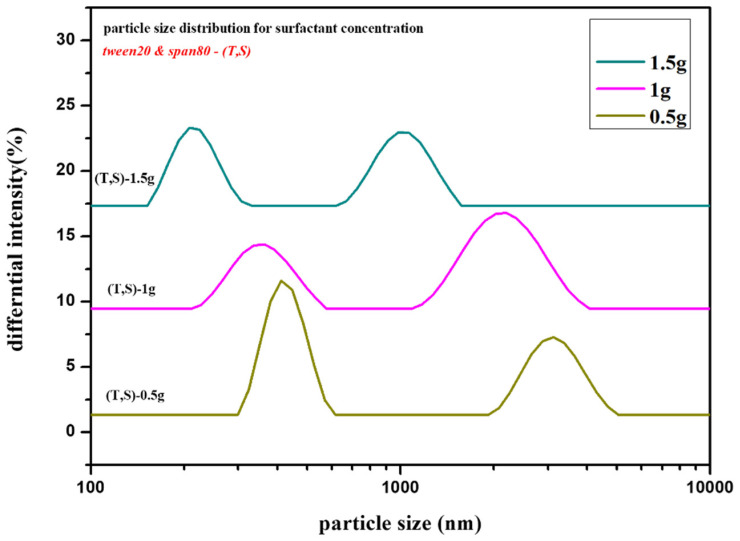
Particle size distribution of different surfactant variations: *S_1_* = 0.5 g, *S_2_* = 1 g, and *S_3_* = 1.5 g.

As shown above, in Figure 5, the peaks associated with a lower concentration (*S_1_* = 0.5 g) of tween 20 and span 80 indicate a larger particle size distribution. A lower surfactant coverage at the droplet interface results in less effective stabilization and more coalescence during particle formation. On the other hand, the particle size peaks for an ideal concentration (*S_2_* = 1 g) of tween 20 and span 80 show a smaller distribution, resulting in improved emulsion stability and reduced size of hollow silica particles compared to lower concentration. Furthermore, compared to the first two concentrations of surfactants, the observation for the concentration *S_3_* = 1.5 g of tween 20 and span 80 shows smaller peaks contributing to the smaller particle size. However, it is crucial to note that excessively high surfactant concentration may also introduce challenges, such as increased viscosity, potentially affecting the hollow structure of silica particles.

In the *OWO* double emulsion process, surfactant concentration (*s*) variations considerably impact droplet size and particle size distribution. After analysis, the observation demonstrates that the surfactant concentration must be balanced to maintain the appropriate size and morphology of droplets and particles. Systematic changes of tween 20 and span 80 surfactant concentration revealed their significant impact on droplet size and particle size distribution. These findings highlight the impact of surfactant concentration on emulsion stability and desirable particle morphology.

### 2.3. Effect of Silica (Water Glass) Content Concentration [x]

After observing the impact on the hexane-to-water glass ratio in the primary O/W emulsion and surfactant concentration in both primary and double emulsion, our finding was focused on one of the critical factors affecting the final particle characteristics, namely the water glass (silica) concentration in the water phase. To determine the effect on particle morphology and shell thickness, systematic decreasing concentration variations were applied. Starting with the initial concentration *x_3_* = 8.68% of water glass concentration serving as a reference point, other concentration variations *x_1_* = 4.92% and *x_2_* = 6.09% were investigated. As shown in Table 1, the variations in water glass concentration were investigated to evaluate their impact on the resulting silica aerogel particle shell thickness.

#### 2.3.1. Formation Mechanism

**Figure 6:**
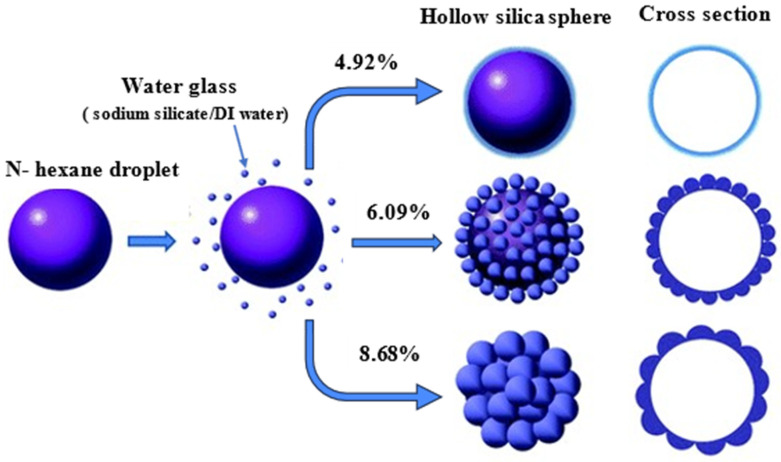
Schematic representation of formation mechanism of hollow silica (SiO_2_) aerogel by varying silica content concentration (*x*).

**Figure 7:**
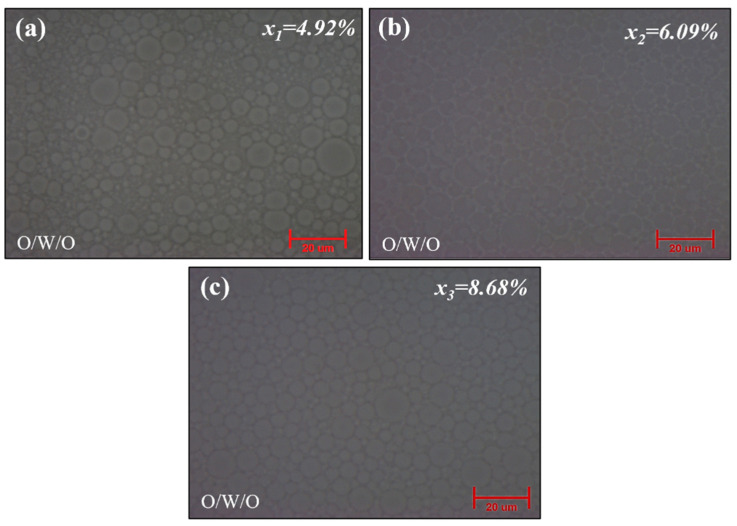
Optical micrographs of silica concentration variations (*x*): (**a**) *x_1_* = 4.92%, (**b**) *x*_2_ = 6.09%, (**c**) *x*_3_ = 8.68%.

The formation mechanism of the hollow silica aerogel particle via the O/W/O double emulsion method is carefully influenced by silica content variation, as shown below in the schematic diagram in Figure 6. The schematic picture represents the initiation of the emulsion process with only a small quantity of silica precursor at a lower silica concentration of *x_1_* = 4.92%, resulting in the formation of thin silica shells during gelation. This observation is supported by the corresponding microscopy images in Figure 7a, which show a thin shell structure. As the silica content rises to *x_2_* = 6.09%, the thickness of the silica shell increases slightly due to the increased availability of precursor molecules. The microscopy examination shown in Figure 7b confirms this depiction, revealing a slight increase in shell thickness. Notably, an increase in shell thickness is observed at an initial silica concentration of *x_3_* = 8.68%, emphasizing the direct relationship between silica content and shell thickness. From Figure 7c, microscopy images show a more substantial and well-defined shell structure. In precisely obtaining hollow silica aerogel particles with desired properties for use in lightweight materials and insulation, this formation mechanism clarifies the controlled modulation of silica content as a crucial factor in adjusting the thickness of the silica shell.

A simple mathematical model can be used to describe the development mechanism of hollow silica aerogel particles as well as their relationship with silica concentration. Based on the data, the following linear relationship may be assumed:

*T* = *kC* + *b*

where the following definitions hold:

*T* is the thickness of the silica shell.
*C* is the initial silica concentration.
*k* is a proportionality constant.
*b* is the intercept, representing the initial shell thickness when the silica concentration is minimal.

The results from Figure 7a–c, which demonstrate an increase in shell thickness with increasing silica content, provide support for this linear model. This relationship highlights the controlled variation of silica content as a critical component in adjusting the shell thickness, allowing for the exact modification of hollow silica aerogel particles for certain applications.

#### 2.3.2. Morphological and Structural Study

As we discussed above and shown in Figure 7, microscopy analysis observed a significant correlation between the silica concentration and the silica shell’s thickness in double emulsion droplets. As compared to Figure 7c, Figure 7a,b show a noticeable decrease in silica shell thickness, which suggests a thin shell structure. This result aligns with an established principle in the field, where a lower water glass concentration corresponds to a thinner shell because of the limited availability of silica content during the gelation process.

**Figure 8:**
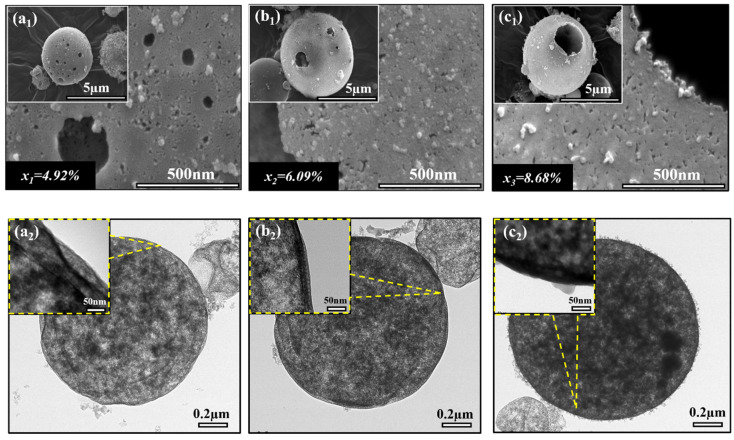
SEM and TEM analysis of silica aerogel particles with silica concentration variations (*x*): (**a_1_**,**a_2_**) *x*_1_ = 4.92%, (**b_1_**,**b_2_**) *x*_2_ = 6.09%, (**c_1_**,**c_2_**) *x*_3_ = 8.68%.

On the other hand, the complex structural variations of hollow silica aerogel particles were examined using scanning electron microscopy (SEM) and transmission electron microscopy (TEM). As shown in Figure 8(a_1_,a_2_), SEM imaging and TEM analysis revealed silica particles with a relatively thin shell at *x_1_* = 4.92%, indicating minimal precursor availability during emulsion and gelation. This thin shell structure of silica aerogel particle agreed with the microscopy analysis of double emulsion droplets, as shown in Figure 7a, providing a thorough understanding of the initial phases of particle formation. 

When silica concentration was increased to *x_2_* = 6.09%, the SEM image, as shown in Figure 8(b_1_), showed the formation of a thicker shell as compared to the previous concentration around the hexane core. TEM analysis from Figure 8(b_2_) shows a noticeable increase in shell thickness, indicating the presence of silica precursor and thus enhanced shell thickness. However, from Figure 8(c_1_), the spherical silica particle’s surface and thickness show an improvement compared to the first and second concentration variations. For confirmation, from Figure 8(c_2_), TEM analysis demonstrated an additional and precise increase in shell thickness at the initial concentration *x_3_* = 8.68%. These results highlighted the direct impact of silica content on the shell thickness. Maintaining the initial concentration of silica content is essential for improving the properties of hollow silica aerogel powder. 

## 3. Conclusions

In this study, the hollow silica aerogel particles were synthesized by a double emulsion (oil-in-water-in-oil, O/W/O emulsion) method using water glass (water phase) and N-hexane (oil phase) as well as hydrophilic (tween 20) and hydrophobic (span 80) emulsifiers. This work highlights the essential parameters influencing the morphology and properties of hollow silica aerogel particles by varying the N-hexane-to-water glass [*OW*] ratio (*r*), silica content concentration (*s*), and surfactant concentration (*x*). The X-ray diffraction (XRD) pattern indicates the presence of amorphous peaks without any impurities. From optical microscope analysis, primary O/W emulsion ratio variations suggest that the reproducibility of droplet formation is improved by an equal distribution of water and oil phases, and variations in surfactant concentration (*s*) show a considerable impact on droplet size distribution. In addition, detailed investigations using zeta potential measurements reveal that systematic changes of tween 20 and span 80 surfactant concentrations impact particle size distribution. Scanning electron microscopy (SEM) and transmission electron microscopy (TEM) analyses suggest that reducing the amount of hexane (oil) impairs the formation of the hollow structure, emphasizing the importance of the primary O/W emulsion ratio (*r*) in determining particle morphology, and maintaining the initial concentration of silica content (*s*) is essential for improving the properties and shell thickness of hollow silica aerogel powder. This study improves the knowledge of the OWO double emulsion approach and provides a versatile and productive approach to producing surface-modified hollow silica aerogel particles with the desired properties for multiple applications.

## 4. Materials and Method

### 4.1. Materials 

The materials used for the synthesis of hollow silica aerogel particles were water glass (silica content: 28–30 wt.%, SiO_2_/Na_2_O = 3.4:1, Young Il Chemical Co., Ltd., Incheon, Republic of Korea), n-hexane (95%, Samchun Pure Chemical, Seoul, Republic of Korea), polyethylene glycol sorbitan monolaurate (tween 20, Sigma Aldrich, St. Louis, MO, USA), sorbitan monooleate (Span80, Junsei Chemical Co., Ltd., Tokyo, Japan), acetic acid (99.5%, Samchun Pure Chemical, Pyeongtaek, Republic of Korea), ethyl alcohol (95.0%, Samchun Pure Chemical, Republic of Korea), and hexamethyldisilazane (HMDS, 98%, Samchun Pure Chemical, Republic of Korea).

### 4.2. Method 

#### 4.2.1. Preparation of Oil-in-Water-in-Oil (*OWO*) Double Emulsion

The hollow silica aerogel particles were synthesized from a double emulsion (oil-in-water-in-oil, O/W/O emulsion) and subsequent emulsion polymerization process. Two-step emulsifications were used to prepare the O/W/O double emulsions. In the first step, the primary emulsion (oil-in-water, O/W emulsion), which included n-hexane droplets dispersed in the water glass, acetic acid, and ethyl alcohol solution, was homogenized at the speed of 6000 rpm for 10 min using an IKA Ultra Turrax homogenizer (T25:S25D-10G-KS, IKA Werke, Konigswinter, Germany). The temperature was fixed at 20 °C during the emulsification process. Acetic acid and ethyl alcohol in an aqueous phase were used for the hydrolysis of water glass and gelation of silicic acid, respectively. The primary emulsion was stabilized using a hydrophilic emulsifier, tween 20. Three different ratios (*r*) of water glass solution to n-hexane (20:60, 30:50, and 40:40) were studied to establish the effect of the ratio (*r*) on the size and morphology of hollow silica aerogel particles.

The second step for the O/W/O double emulsion was the emulsification of the primary O/W emulsion into n-hexane. The primary O/W emulsion was slowly added to n-hexane containing span 80 and then emulsified using the homogenizer at 6000 rpm for 10 min. The primary emulsion to n-hexane of double emulsion ratio was fixed at 40 (water glass):40 (n-hexane):40 (n-hexane). The concentrations of surfactants (tween 20 and span 80) and the water glass solution are given in Table 1.

#### 4.2.2. Preparation of Hollow Silica Aerogel Powder

**Figure 9:**
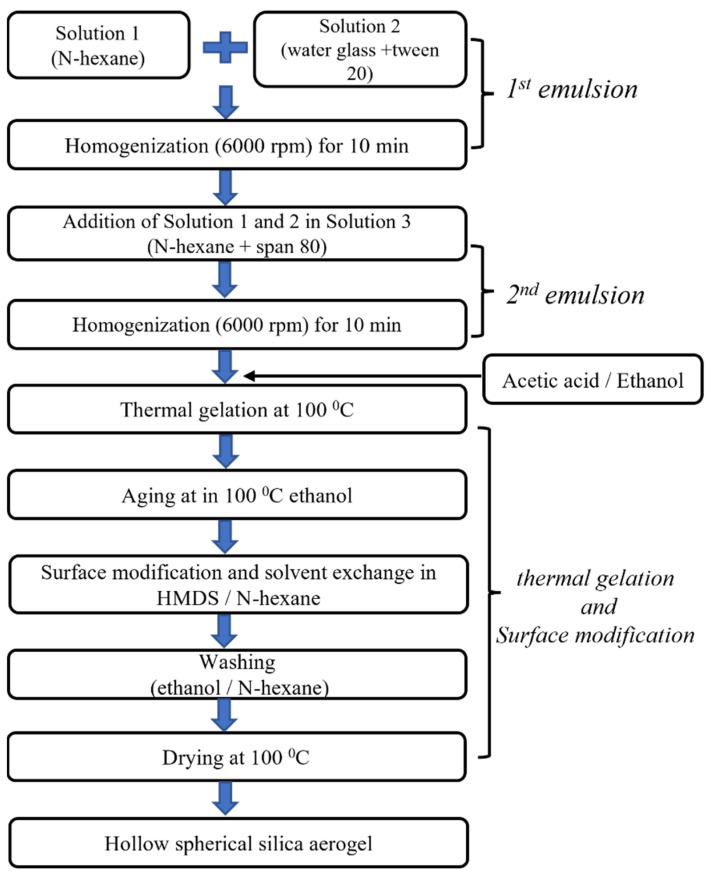
Experimental flow chart for the synthesis of hollow silica aerogel particles by *OWO* emulsion.

Hollow silica aerogel particles were synthesized from the O/W/O double emulsion; each particle consisted of an n-hexane core (droplet) and a water glass shell that was dispersed in n-hexane. The O/W/O double emulsion was heated at 80 °C, and the temperature was maintained for 90 min to induce the hydrolysis and gelation of the water glass shell. Then, core (hexane)–shell (silica gel) particles were immersed in 150 mL ethyl alcohol for solvent exchange, possibly resulting in a hydrogel-to-alcogel transition. The silica wet gel shell was chemically modified in 150 mL of 20% hexamethyldisilazane (HMDS)/n-hexane solution at 80 °C for 3 h with magnetic stirring at the speed of 100 rpm. The silylated silica wet gel was washed in an ethyl alcohol/n-hexane solution to remove any remaining surface modification agents and reaction products. Then, silica wet gel spheres containing n-hexane cores were dried at 100 °C under ambient pressure for 1 h. The experimental flow chart for the synthesis of hollow silica aerogel particles is shown below in Figure 9.

#### 4.2.3. Characterization

The size and distribution of n-hexane droplets in the O/W emulsion and n-hexane core/silicic acid shell droplets in the O/W/O emulsion were observed by optical microscopy (OLYMPUS-BX53M, Tokyo, Japan). The phase identification of the hollow silica aerogel was performed using X-ray diffraction (XRD-Smart Lab SE from Standard Analysis Research Institute, Inha University) analysis. A particle size analyzer (Zeta Potential Analyzer-ELS-Z, Standard Analysis Research Institute, Inha University) was used to determine the particle size distribution of the obtained hollow silica aerogel powders. Scanning electron microscopy (SEM-Hitachi S-4300, Sustainable Energy Components and Materials Core Research Support Center, Inha University) was used to examine the morphology of hollow silica spheres. Transmission electron microscopy (TEM-CM200, from Standard Analysis Research Institute, Inha University) revealed the hollow structure of silica particles. 

## Figures and Tables

**Table 1:** Optimization conditions for the fabrication of hollow silica aerogel particles.

Sr. No	First Emulsion		Second Emulsion	Surfactant		Silica Content (%)	RPM	Time
	Water Glass (mL)	N-Hexane (mL)	N-Hexane(ml)	Tween 20 (g)	Span 80 (g)			
1	20	60	40	1	1	8.68	6000	20 min
2	30	50						
3	40	40						
4	40	40	40	0.5	0.5	8.68	6000	20 min
5				1.5	1.5			
6	40	40	40	1	1	4.92	6000	20 min
7						6.09		
8						8.68		

## Data Availability

All data and materials are available on request from the corresponding author. The data are not publicly available due to ongoing research using a part of the data.
